# Implementation of a Remote Instrumental Music Course Focused on Creativity, Interaction, and Bodily Movement. Preliminary Insights and Thematic Analysis

**DOI:** 10.3389/fpsyg.2022.899381

**Published:** 2022-05-20

**Authors:** Andrea Schiavio, Luc Nijs

**Affiliations:** ^1^Centre for Systematic Musicology, University of Graz, Graz, Austria; ^2^Institute of Psychoacoustics and Electronic Music (IPEM), Department of Art, Music, and Theatre Sciences, Ghent University, Ghent, Belgium; ^3^CORPoREAL, Royal Conservatory of Antwerp, Antwerp, Belgium

**Keywords:** music learning, musical creativity, musical interaction, music and movement, remote learning

## Abstract

In a newly designed collaborative online music course, four musical novices unknown to each other learned to play the clarinet starting from zero. Over the course of 12 lessons, a special emphasis was placed on creativity, mutual interaction, and bodily movement. Although addressing these dimensions might be particularly challenging in distance learning contexts, a thematic analysis of semi-structured interviews with the learners revealed how the teaching approach proposed has generally facilitated learning. Qualitative findings highlight the importance of establishing meaningful relationships with the musical instrument as well as with other students to build musicality, and of the interplay between creativity and control in individual and collective music-making activities. We suggest that remote music tuition with a small group can be a valuable resource to start learning music and that a creative, collaborative, and movement-based approach can contribute to musical growth.

## Introduction

When the COVID-19 pandemic was at its peak, many school activities were urged to adopt e-learning platforms to avoid physical contact and close interaction between students and between students and teachers (Hodges et al., 2020; Aurini and Davies, 2021). A wealth of recent studies has reported that many learners might have been exposed to considerable psychological stress and associated learning shortfalls when transitioning from live to remote education in such a dramatic period (Cao et al., 2020; Engzell et al., 2021). As noted by Habe et al. (2021), settings such as music and sports, in which human presence and physical contact are seen as fundamental dimensions for the learners’ flourishing, have suffered significantly (Antonini Philippe et al., 2020; Mehrsafar et al., 2020; Spiro et al., 2021; Woodford and Bussey, 2021). For this reason, exploring in detail how students may or may not benefit from distance activities in contexts such as instrumental music education can be of paramount importance to improve our current knowledge on both domain-specific and general pedagogical issues (Biasutti et al., 2021; de Bruin, 2021). Consequently, a thorough examination of the weaknesses and strengths associated with the different manifestations of distance learning can help improve existing pedagogical settings based on virtual interaction, stimulating new research, theories, and practical insights that can be applied to a variety of contexts in which the extensive use of technological resources for remote learning is necessary (see also Dammers, 2009; Cayari, 2011; Burrack, 2012; Daffern et al., 2021; MacDonald et al., 2021).

The present article contributes to this line of research by reporting on the verbal descriptions and personal insights of four adult, musical novices^1^ who were invited to enroll in a newly created 12-week online music course dedicated to learning how to play the clarinet. As we shall see later in more detail, the course—taught by author LN—was developed with the precise intention to foster an inclusive pedagogical experience based on creativity, interaction, and bodily movement in the context of remote learning. These latter aspects are often deemed crucial in face-to-face lessons and are generally seen as fundamental factors to facilitate the development of musical skills and a meaningful pedagogical experience in a variety of settings (Burnard, 2002, 2016; Odena, 2018; Addessi, 2020; Schiavio et al., 2021b). However, it has been argued that such categories are not always given the attention they deserve in many musical learning contexts (see Persson, 1994; Rostvall and West, 2003; Borgo, 2007); furthermore, addressing creativity, interaction, and bodily movement could also be more difficult in group distance learning contexts when compared to more traditional musical settings (e.g., one-to-one tuition), given the inherent differences between physical and virtual presence (Pike and Shoemaker, 2013; Hash, 2020; Obrad, 2020; Willatt and Flores, 2021). And indeed, an important aspect of the present study is that our participants started learning to play the clarinet in a remote environment right away. This differs from other studies focused on the pedagogical transformation faced by many students who have suddenly been confronted with the transition from face-to-face to online music tuition due to the COVID-19 pandemic in music as well as other disciplines (e.g., Gillis and Krull, 2020; Hoss et al., 2021).

The general aim of this research is to report and contextualize the personal perspectives of the learners involved in this course on how these three categories (again, creativity, interaction, and bodily movement) can be experienced in remote learning, offering in turn new considerations that may be relevant for implementing similar programs in the future. We proposed in our course several creative musical situations and collaborative learning experiences based on movement and action, which the participants were invited to engage with, reflect on, and comment upon. We chose to work with a group of four participants as previous research highlighted how group creativity—i.e., a set of people working together to produce unique and meaningful outcomes and ideas (Hoever et al., 2012)—might be enhanced in small- and medium-sized groups, promoting an optimal balance between individual and collaborative behaviors (De Rosa et al., 2007). Music provides an ideal context in which this can be put to test, as learning to play an instrument involves a good deal of solo (i.e., individual practice) as well as joint activity (i.e., musicking with teachers and peers; Zhukov, 2012; Reid and Duke, 2015; Creech and Hallam, 2017; Längler et al., 2018).

In what follows we briefly frame the three main categories at the heart of this study (creativity, interaction, and bodily movement) relative to music and music pedagogy research, providing specific examples of relevant activities carried out during the course. Having done so, we report on and contextualize our preliminary qualitative findings in the sections that follow.

### The Main Ingredients: Creativity, Interaction, and Bodily Movement

While defining *creativity* with precision remains highly problematic (see, e.g., Sternberg and Lubart, 1996), this phenomenon might be conceived of as a capacity to think “outside the box” and to act accordingly, giving rise to items (e.g., musical phrases, learning exercises, etc.) that are at the same time innovative, surprising, and task efficient (see Boden, 1990, 2010). There is excellent work in music performance and music education research addressing such a topic from an interdisciplinary perspective (see Deliège and Wiggins, 2006; Running, 2008; Burnard, 2012, 2013; Cook, 2018; Barrett et al., 2021). In his book *Musical Creativity Revisited*, for instance, Odena (2018) illustrates a rich variety of findings from systematic investigations conducted across several countries, offering theoretical and practical insights that concern both the concept of (musical) creativity itself and the notion of pedagogy of creative collaboration. By considering the impact of reciprocal interaction for creative musicking in diverse educational settings, the author refers to creativity as “the development of a musical output that is novel for the individual(s) and useful for the situated musical practices” (Odena, 2018, p. 51).

Inspired by such insights, the course was organized to let students find their own creative path through bodily/instrumental exploration as well as through collaborative work. To do so, throughout the 12 lessons not only did the teacher propose “guided” creative activities, but also encouraged participants to explore different nuances of the group setting to find an optimal, constructive synergy. An example of a creative exercise put forward by the teacher was to turn a *haiku*^2^ into music: students were given, in pairs, a poetic component and were asked to collaboratively “translate” it into music. This task was followed by a performance in which each student showed his or her interpretation and then by a moment of joint reflection, where all participants could comment on both improvisations (one per pair) and describe verbally *how* the music was generated. Students were also asked to come up with their own creative ideas and present them to the group in various ways. They were given the opportunity to propose specific topics and address them collaboratively.

In one of the final lessons of the course, for instance, students almost unanimously suggested to work on sound production, with a specific focus on low notes and tonguing in relation to tempo. Both aspects were then explored through a creative exercise based on an integration of hearing and tonguing, which was jointly designed on the spot: one student was first asked to invent a melodic pattern using three low notes; then, another participant was invited to rapidly imitate this pattern without thinking too much. This also led to a discussion between students concerning the quality of the original pattern and the accuracy of the imitation. Understanding how similar opportunities for creative musical thought and action arise and are experienced in a context where face-to-face interaction is hindered can be particularly important for shedding new light on how creative teaching and learning may unfold in similar participatory settings (see Haddon, 2016).

This last point speaks of the role of *interaction* for musical skill acquisition—the second dimension we wished to point up in the clarinet course we have developed. There is already a vast literature highlighting how forms of learning involving more students at the same time can foster positive learning experiences (see, e.g., Burnard et al., 2008; Gaunt and Westerlund, 2013a; Hanken, 2016; Nielsen et al., 2018; Schiavio et al., 2019, 2020a). In these works, the profound connection between creativity and collaboration is also often emphasized from a pedagogical perspective, complementing contributions that explore such a phenomenon in domains, such as management, economy, sport psychology, and music-making (Amabile, 1983; Sawyer, 2003; Perry-Smith, 2006; Gesbert et al., 2022; van der Schyff and Schiavio, 2022). The context of teaching instrumental music is therefore, in a sense, unique when it allows to combine group creativity and collaborative learning in a seemingly natural way. Indeed, despite the Western traditional focus on individual practice and one-to-one education modalities (see Gaunt et al., 2012; Hallam and Bautista, 2018; Lehmann and Jørgensen, 2018), much learning occurs in groups, and peers often make music together (both formally and informally), acquiring and developing their skills in the process (see Cope, 2002; King, 2008; Gaunt and Westerlund, 2013b; Forbes, 2020). Two recent empirical studies, for instance, have demonstrated that novices who learn to play the piano or the drums with another peer can produce musical performances as accurate as those generated by novices learning by themselves, suggesting that the individual forms of learning can be complemented with more participatory approaches without disrupting the learning trajectory of the student (Schiavio et al., 2020b, 2021b). But as such work focuses on peer interaction unfolding in mutual presence, the possibilities that a collaborative approach holds for remote learning and the experiences it gives rise to students remain to be further addressed.

For this reason, during the course, participants were actively encouraged to work in pairs or all together, exploiting the potential of technology (e.g., Zoom) in different ways. For example, in each lesson, Zoom “breakout rooms” were used with a variety of intentions and purposes: students could chat or greet each other to build a sociable atmosphere at the beginning, then could discuss possibilities for creating new exercises, practice a difficult passage jointly, or execute a particular task which they were mutually responsible for. For example, as will also be discussed later, the teacher could invite students to generate a musical phrase (e.g., a simple melodic or rhythmic theme) or a linguistic phrase (i.e., a series words constituting a grammatical unit), which could then be examined and transformed from multiple artistic perspectives: how to make the musical phrase more expressive? What fingering to use? Or, how to give the linguistic sentence a musical form? Should we be inspired by the meaning of the sentence, or by its phonemic? On such occasions, the teacher could explicitly illustrate the type of task required (e.g., “please create a melody that reflect how this phrase sounds to your ears using the two notes we learned today”) or be deliberately ambiguous (e.g., “be inspired by this sentence and create a sonic pattern out of it”) letting the pairs discuss options, improvise, or compare ideas. These examples show how the group was constantly invited to explore different possibilities to achieve a concrete result and use information from others to transform and reshape the ideas initially generated^3^.

The focus on creativity and collaborative learning discussed above was associated in the course with the role of the body and of *bodily movement*—a category that has been thoroughly examined in a range of musical contexts (see, e.g., Davidson, 2002; Bowman, 2004; Doğantan-Dack, 2006; Hubrich, 2016; Leman and Maes, 2016; Tanaka and Donnarumma, 2019). In these regards, recent *embodied* approaches to musical experience and to human cognition more generally have significantly reshaped the conceptual landscape in which body, action, and movement are studied and understood (Varela et al., 1991; Borgo, 2005; Gallagher, 2005; Leman, 2007; Chemero, 2009; Gallagher, 2017; Reybrouck, 2020; van der Schyff et al., 2022). Very generally speaking, the embodied standpoint holds that the human mind is continuous with (i.e., partly constituted by) body and action rather than being a separate^4^ category from it. In other words, where traditional views often equate mind to (the abstract laws and algorithms supporting) information processing (see, e.g., Gardner, 1985), or identify it to neural structures (see, e.g., Lewis, 1966), scholars working from an embodied perspective see mental life as a property of a brain–body system in action. This means that categories such as movements, actions, or gestures can be understood as cognitive tools on their own, which work in tandem with the brain and with other ecological resources to solve problem, think, feel emotion, communicate, and act intelligently (see Sheets-Johnstone, 1999, 2010; Shapiro, 2011; Wilson and Golonka, 2013). In the context of instrumental music education, however, there is a risk that the body can only be considered as an input device that receives information from the world to trigger practical responses or positive changes in learning rather than as a constitutive part of the human mind (see van der Schyff et al., 2016 for discussion). This trend arguably resonates with situations in which students (are asked to) imitate the teacher’s actions and movements to improve musicianship and instrumental technique: without real motor autonomy and independence to generate movements, however, we suggest that the body may not become a significant (cognitive) resource for the student’s musical flourishing and participate in learning and musicking with its full potential (see Schiavio and van der Schyff, 2018). With this in mind, it has been argued that a larger variety of bodily movements—beyond the necessary ones to play an instrument—might be an integral part of a meaningful learning experience (Nijs, 2017; Bremmer and Nijs, 2020). Free bodily movement can thus be seen as vital for musical learning and human musicality more generally, as it provides a natural way of thinking musically, in turn shaping posture, personal style, and instrument-specific actions (Juntunen, 2016; Nijs, 2019).

Building on such insights, students attending our course were invited to use their bodies creatively and freely in different ways. For instance, they were often asked to explore the degrees of freedom in their joints while playing music (e.g., moving the feet freely during an improvisation). This helped them create a better awareness of the connections between body, instrument, and environment. In other moments they were invited to explore broad lateral movements as well as the multiple possibilities for phrasing and breathing those such movements gave rise to. The newly discovered motor configurations were then contextualized, re-explored, hybridized and, if necessary, transformed on the spot, depending on the task, their mood, taste, or on other variables defined in advance by the teacher. Importantly, while similar movement-inspired individual work was carried out by the learners between the lessons, much exploratory-motor activity was also done when meeting together. This stimulated critical discussion and reflection, also providing each learner with an opportunity to put themselves in the shoes of others when certain movements are performed (or simply explored) in specific moments.

In all, each lesson of the course gravitated around the themes of creativity, interaction, and bodily movement, broadly conceived, whereby doing and reflecting were always integrated. By examining how the first musical-learning experiences of our participants developed in this remote learning environment, we explored how our musical course was experienced by novice learners through an examination of the verbal reports they offered in two individual interview sessions. The present research thus aims at providing concrete examples of how students engage with an online music course that was specifically designed to focus on creativity, interaction, and bodily movement. We expected that not only these categories would be experienced in a positive way by the students, but also that would be conceived of as an essential aspect of their learning trajectory, regardless of the online medium through which the course was offered. In what follows, we first describe the methods of the study, with a focus on the rationale guiding the analytical procedure we adopted. We then report on the qualitative data emerged from the interviews conducted with each participant and discuss how these findings can provide richer understandings of the creative, collaborative, and movement-based aspects at the core of the program, and how these can be helpful for future research and practice.

## Materials and Methods

The present research is part of a larger collaborative investigation exploring how non-musicians at the initial phases of instrumental musical learning can benefit from collaborative online resources and how a creativity-oriented music course can be designed accordingly. In the present study, we focus on the subjective learning experiences that participants reported during two sessions of semi-structured interviews. The qualitative data reported here have been specifically analyzed considering the following research questions: How does a group of novice adult learners experience a remote instrumental musical course which emphasizes creativity, interaction, and bodily movement? How can this content be optimized and delivered in such a setting? The study was carried out in accordance with the Declaration of Helsinki and the Code of Ethics and Conduct of the British Psychological Society. All participants were informed about each task and procedural step of the study—including data anonymization—and provided written informed consent. As the study was formally conducted in Belgium, ethical approval was not necessary, following Belgian Laws for research practice (see. “May 7, 2004 Law concerning experiments on the human person”).

### The Course

A 12-lesson course was designed by both authors to facilitate instrumental music learning during the COVID-19 pandemic that took over during 2020 and 2021. The course involved the development of basic instrumental (e.g., posture, fingering, embouchure, and breathing) and musical (e.g., playing rhythms and melodies and hearing) skills based on playing by ear and improvisation. Although such a description may also apply to more traditional teaching approaches, particular attention has been paid to creativity, interaction, and body movement, intended both as learning objectives and as elements on which to develop one’s musical skills. Every week, participants took part in a 1-h, collaborative music lesson *via* Zoom. Next to being driven by the main “ingredients” discussed above, we were inspired by a set of pedagogical principles.

The first principle may be labelled as *From Sound to Sight*, whereby new content was always introduced aurally (e.g., Kohut, 1985; McPherson and Gabrielsson, 2002). Gradually, lesson after lesson, theory was added, for example, to explain how basic musical scales are formed. However, traditional notation on a staff was never used. Another guiding principle was named *Exploration and Experimentation* (see Borgo, 2007; Moreira and Carvalho, 2010;). Participants were always invited (individually or collectively) to explore and experiment with the new material being presented during class. This allowed them to playfully engage and familiarize themselves with the building blocks of the lesson content. For example, before learning a song, several creative, movement-based, and collaborative activities based on the rhythm or notes of the song itself were introduced. This involved improvising on such a musical material, for instance, inventing new melodies and variations on the lyrics. Also, *Multimodality* was an important design principle of the lessons (e.g., Hammel, 2003; Nijs, 2018). This implied that, after a first phase based on the first pedagogical principle described above, musical content was always approached through verbal, visual, and bodily modalities. Consider here how tonguing (i.e., hitting the reed with the tip of the tongue to articulate notes) was introducing through translating self-invented verbal phrases into a “tu-language” (e.g., “hello, how are you?” becomes “tu tu, tu tu tuu?”) and then played by repeating a single note. The rhythm was then combined with movements (e.g., lateral movement with the clarinet to suggest air stream, or stepping exercises to introduce meter). Subsequently, different notes were used to differentiate ways of expressing the verbal phrase. Students were often invited to do this in dyads, inventing “musical dialogues” on the spot. Another pedagogical principle may be labeled as *Non-Linearity*, in the sense that lesson content was adapted in real time to the initiatives and needs of the participants instead of strictly following a predetermined series of consecutive steps. For example, at one point, one of the students asked to learn to play a theme from “Star Wars.” As a result, novel notes and rhythms were introduced, which in a more traditional approach would only be learned later in the curriculum.

**Figure 1:**
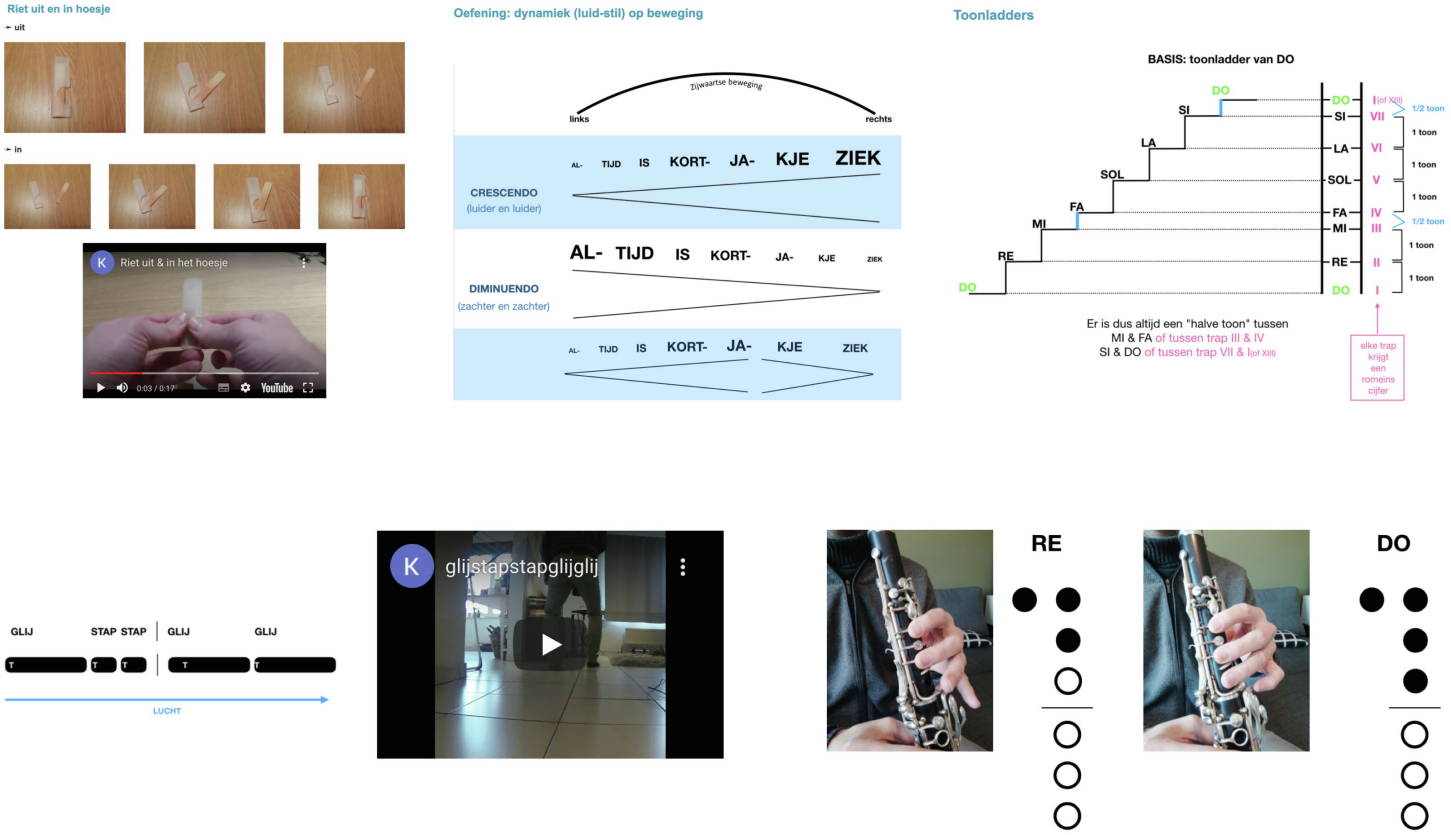
Supporting material accessible *via* a dedicated website. This involved videos on different aspects of the learning process, visual representation of tasks (e.g., rhythm for improvisation) or learning content (e.g., new note fingering), as well as sound-files and other didactic material. Screenshots taken from private Youtube channel “Klarinetles”: **(left)** Luc Nijs, “Riet uit and in hoesje [reed in and out cover]”, YouTube video, 0:18, 01/04/2021; **(middle)** Luc Nijs, “glij stap stap glij glij [glide step step glide glide]”, YouTube video, 0:12, 01/15/2021.

It should also be noted that all lessons were supported by digitized learning content, made accessible through a dedicated website. These included videos on technical aspects of playing the clarinet, explanations of content that was addressed during the lesson, as well as visual aids (see Figure 1).

Finally, to support communication with the teacher beyond the lesson, the platform Flipgrid®^5^ was used. This is a website and app that allows teachers to facilitate the students’ engagement, discussions, and collaboration by offering them an opportunity to post tasks (e.g., a recording of their performance) and questions (e.g., a particular issue concerning the fingering of a scale) using videos and other interactive material.

### Participants

Four adult non-musicians, unknown to each other, took part in the study (one woman, three men; mean age = 36.75 years; *SD* = 7). They were recruited after a call was circulated through social media (i.e., Facebook). Participants did not have prior experience with playing an instrument or with taking music lessons—even informally. They chose to enroll for different reasons: out of curiosity, to engage in learning something new, or envisioning to play in a wind band. Next to the lessons being tuition free, they were offered a financial compensation for taking part in the study. To ensure anonymity, in what follows participants are identified with a number from 1 to 4. The sample size is compatible with other published research focused on group music-making and learning (e.g., Schiavio and Høffding, 2015; Ridout and Habron, 2020) and was chosen because it was considered particularly functional to the type of course developed (as mentioned above).

### Procedure

After lesson 4 and 12, participants were interviewed individually through Zoom. A protocol was designed by the authors to guide the interviews and let the participants cover a variety of topics in a systematic way (see Appendix). As each interview (*n* = 8, in total) lasted between 34 and 58 min for an average of 48.1 min, there was sufficient time to explore in detail the different topics. Semi-structured interviews are well poised to gain access to the lived experience of participants involved in a collaborative task and have been extensively adopted in musical research (see, e.g., Biasutti, 2018; Crawford, 2019). As each participant was interviewed two times, we use the letters “A” and “B” to differentiate between the two interviews, where A refers to interviews conducted after 1 month (after lesson 4), and B to the final interviews (after lesson 12).

### Data Analysis

**Figure 2:**
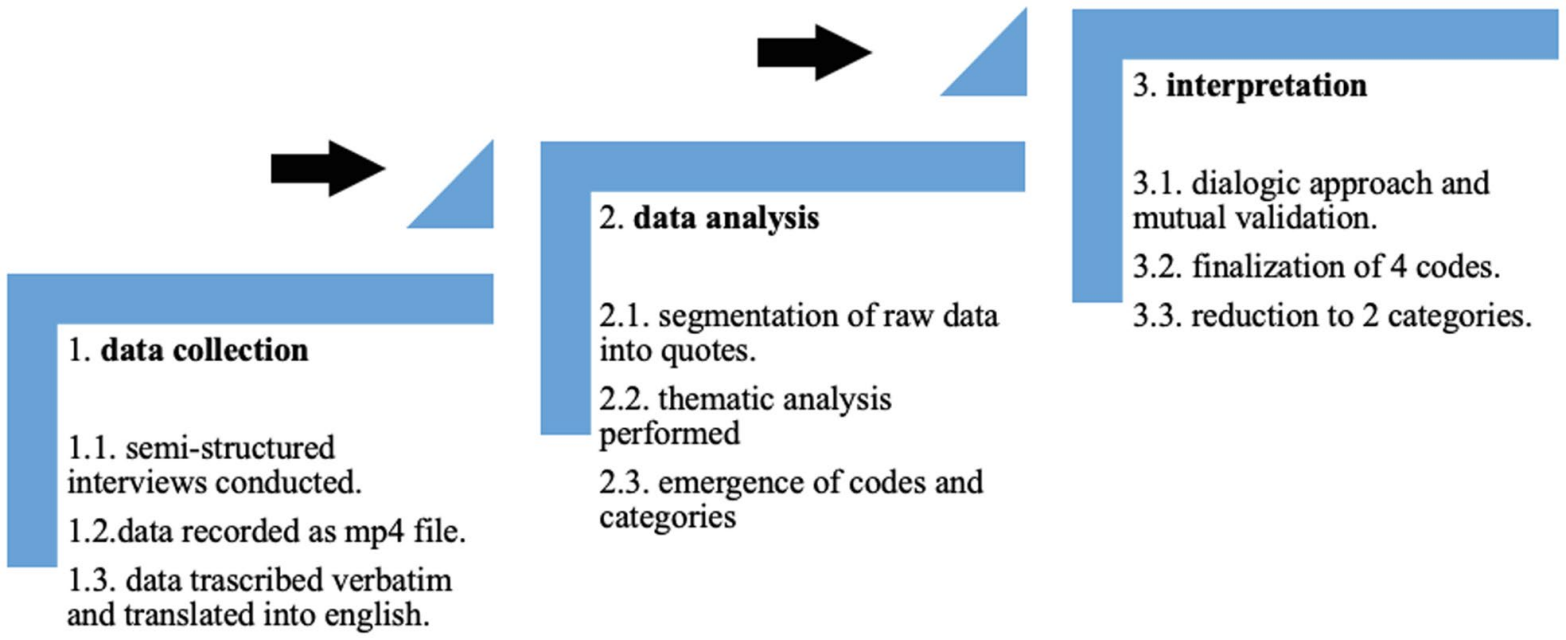
The different steps of data analysis.

All interviews were recorded as an mp4 file, transcribed verbatim, translated into English by the second author, and systematically organized for the analysis (i.e., they were segmented into separate quotes and merged into one Word document). The material was then analyzed using a grounded theory approach. The latter’s interpretative nature puts the analyst’s personal reflections at the heart of the coding processes, such that findings entail an explicit encounter between the participants’ constructs and the researcher’s theoretical sensitivity (Glaser, 1978; Birks and Mills, 2015). The small group size combined with the close interaction that interviewer and interviewees experienced during the course ensured a mutual exchange of insight during the interviews consistent with the grounded approach we used. Meaningful themes emerging from the data were organized according to specific codes and categories, and different solutions and interpretations were discussed by both authors. The analytical process was initiated by author AS and then verified by author LN. The latter made sure that quotes initially attributed to a particular code could not be attributed to others and confirmed whether the codes generated by AS were broad enough to capture the range of ideas expressed by the quotes. Such an approach based on mutual feedback gave rise to a total of four codes, which were then reduced to two main categories. Each step of the analytical process is depicted in Figure 2.

## Findings

Here, we report and comment on the students’ experiences of participating in the course through verbal accounts extracted from the interviews. In doing so, we contextualize each quotation considering the course’s main themes, thereby providing a rationale for discussion that follows. As mentioned above, each quote is accompanied by a number (1–4) and a letter (A or B), indicating participant and time of the interview, respectively.

### Building Musical Connections

#### Knowing the Instrument

Starting a completely new activity as an adult is not an easy task and learning music from scratch is no exception. As this venture is intrinsically associated with the kind of relationships students come to develop with their own instrument, it is very important to examine how the first interactions with the latter may feel:

“I have the feeling that the instrument suits me, and that it is something pleasant. But I do not have the feeling that it’s an ‘extension of my arm’ or something like that… it’s not my own. Still a strange instrument where occasionally I can get something pleasant out of it. But I do not have the feeling that it’s my own, that instrument.” (2, A)

This initial difficulty might be discouraging as it can be manifested in several ways: For example, as we are told by another participant: “in the first few lessons I […] had a lot of trouble blowing through the clarinet” (3, A). However, said issues might be mitigated when put into a broader learning context, where enthusiasm and excitement can inspire the student to develop novel ways to engage with the instrument. Consider the following statements, collected after the first four lessons, by two other participants:

“I do not like doing things online like this. I really do not like doing that. I’m someone who prefers to talk face to face with people. So, I expected to have more trouble with that. But the first lessons actually went pretty smoothly, I think. It also amazes me how quickly you learn things on the clarinet anyway.” (1, A)

“There are two things that I am positive about. First, just the fact that I’m learning something new. It’s been a while. And something not directly in my comfort zone or field of expertise. That in itself is nice, and something I’ve been thinking about doing for some time now. Whether it would be languages or an instrument. So, I think that’s partly where the enthusiasm is situated. I also kind of expected that I was going to […] be intrigued by it. […] I did not expect to find much enjoyment in playing the clarinet or any such instrument. I’m pleasantly surprised by the fact that I actually do like it.” (4, A)

The quote suggests that it is precisely the uncertainty of learning that could make this activity a springboard for curiosity, challenge, and personal development. For example, several rhythmic exercises were based on the input of the students: As mentioned earlier, on many occasions they were invited by the teacher to generate simple sentences (e.g., “Hello, how are you today?”) and jointly explore how these could be transformed into musical phrases, where, for instance, specific attention was devoted to find what rhythmical patterns could fit the expressive nuances of the sentence. These sentence-inspired, expressive rhythmical forms were then repeated on different notes (e.g., of a pentatonic scale); when doing so, students were also asked to explore different melodic and dynamic solutions to mirror the most salient features of the sentence (e.g., raising pitch at the end of a question, couple loudness to enthusiasm). Students engaged in similar tasks both on their own and together with others, connecting its exploratory dimension to creativity and emotion:

“I notice that if I have to keep doing the same exercises all the time, it gets boring for me. So, I try to challenge myself by learning to play different notes and then I do the exercises on those notes. […] A creative [player] is someone who starts making up notes and tunes from their own feelings. That’s what I call creative playing.” (1, A)

Through time, this can give rise to positive outcomes and changes in how the instrument is experienced:

“A certain attachment [with the clarinet] has emerged. You have the feeling that it has become a bit ‘my’ clarinet, it has become a place of safety. Yes, a very strong bond did develop there. […] But [the clarinet] also feels like an extension, especially at those moments when you feel that you have to start blowing through the clarinet, that you really blow through it, that you no longer feel pressure in the body, then it really feels like an extension, and then you just forget that you are holding that instrument.” (3, B)

“After a while, that clarinet […] I’m not going to say that I have complete control over it, but […] it’s not a strange object anymore. In the beginning that was really the case, then you look at it not even knowing how to put it together yourself.” (2, B)

But what happens when holding the instrument for the very first times? A way to gain familiarity with it might be found in how the musical possibilities offered by the instrument can be explored in total freedom:

“I always start by being a little creative with it, for my own pleasure, and then I’ll try out some sounds that we have not learned yet, playing with my fingers, so that I get something audibly pleasant that makes me want to experiment more with it, before I force myself into an exercise.” (2, A)

The constructive opportunities that emerge from such a creative freedom with the instrument (e.g., students work in dyads, one student makes a rope or stick move in a creative way, the other student plays a musical pattern while imitating the movement of the rope or stick) are expressed in further descriptions from the same participant reported in the final interview, where exploratory activity is associated with free movements:

“I would not tend to stay in a chair. So, I would rather walk around until I had to stand still because, for example, I’m going to have to read a score or re-enact something and have a screen next to me or something. […] I do think that moving around feels rather natural and sitting or standing will feel rather unnatural.” (2, B)

Engaging with the clarinet in a creative and active way, for example, by exploring music—movement associations while improvising, leads participant 3 to offer the following evocative description, where the instrument is described as “friend”:

“To a friend you can tell a lot of things. For example, negative emotions, you can tell them, and then they are channelled. The same with the clarinet. If you feel down, then you are also going to play more instinctively […], somewhat blue, or feed a little more negative emotions, you are going to put a darker sound in it. Then you actually feel like you are entrusting the clarinet with your emotion, and the clarinet is going to translate it. A bit like a friend does. It’s going to translate it, reformulate it, and then you can start doing something with it.” (3, B)

Note how, in this last quote, an intersubjective characterization of the musical instrument emerges even if the kind of activity described was inherently solitary (i.e., playing the clarinet alone). In all, the statements reported here suggest that despite possible initial problems, there is an important tendency to establish a relationship with the instrument that, through movement and creative effort, develops in an effective fashion.

#### Knowing the Group

The intersubjective framing of the last statement brings the discussion to the social dimension of the course. In a way similar to how a relationship with the instrument is built through the course of the program, our participants revealed different attitudes toward each other, which radically shifted as the course develops.

“In the beginning we were [just] four individuals, we did not know each other. But online a kind of connection emerged. It’s very strange, but that’s what happened.” (1, B)

It is important to note how the quote stresses that a sense of social connectedness emerged *despite* the remote setting of the course. Likely, a different context would have facilitated the participants’ cohesion and sense of togetherness. The same concern is voiced by another participant during the first interview:

“There is no group dynamics, or very little. I think that, once you have the group dynamics […] you are going to [learn] faster, on whatever medium. But if you have not been able to create [adequate] group dynamics [due to] a physical distance—then for me […] it’s harder.” (2, A)

Interestingly, in the final interview, the same participant returns to the topic of group dynamics to discuss on how these have been eventually developed remotely:

“I work in the social sector, so I work a lot with people, and I know that group dynamics is something you cannot build on your screen. But still I had the feeling that there was a harmony in the group, and that sometimes a joke could be made, and that there was spontaneity among each other. Nobody was like ‘what kind of person is that’. But it was all about the music and playing together and trying it out together, without hesitation.” (2, B)

In a similar vein, another participant comments:

“[Even if] we never saw each other live, we managed to make a very pleasant connection. I also notice that at the beginning of the lesson we chat with each other, [and we ask, for instance] ‘How was the week?’. [It is] always very pleasant [that] you can share some experiences. And very quickly we managed to make some jokes with each other during the lesson [too]. I was very positively surprised how fast that went to create that bond. That was a very pleasant feeling. And at the same time a very safe feeling.” (3, B).

Although the remote setting may appear not so helpful at first, the group co-created a friendly and safe learning environment. It is therefore important to examine what kind of teaching dynamics helped such a positive outcome to be developed. One strategy the teacher adopted in the course was to use Zoom breakout rooms, and let students interact and play with each other already from the first lessons:

“Especially when you go into ‘breakout rooms’, you are really actively engaged with each other, and then you see each other make mistakes, or give more difficult assignments. And that’s a positive thing for me, because you see you are not the only one struggling with certain things.” (2, A).

**Figure 3:**
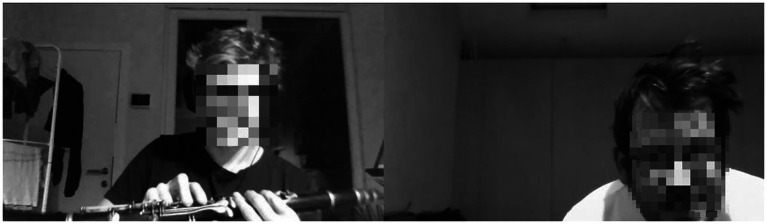
In the breakout room, one student helps the other student by showing a particular fingering solution. Both participants are involved in exploring different fingering solutions.

Not only can such an arrangement provide the student with more learning responsibilities (as the teacher was not there all the time); this can also help novel creative opportunities to be developed directly from the students’ interaction (as illustrated in Figure 3). As the same participant put it:

“The breakout rooms are definitely the most creative aspects, or moments [of the lesson] and I think, yes, every time you learn something new and you succeed, that’s interesting in itself. Suddenly you know an extra note that you did not even know existed. And that in itself is interesting. Or you learn a blowing technique. I remember the moment when you learned that you have to use your tongue to change notes instead of making your breath stop. That’s when you are like: “Ah yes, I actually have not thought that this would be even possible.” So that in itself is such a moment that is grateful.” (2, A)

Participants can thus “learn from each other” (3, B), activating and reinforcing social dynamics through musicking. As such, “you did not have to be afraid of the breakout rooms” (4, B) for the whole duration of the course. An example of this stems from a previously mentioned musical exercise, where learners had to come up with a sentence and then translate that into music. In a number of cases, students were paired and asked to engage in a “dialogue” where (i) they created novel sentences one after the other, (ii) discuss the content of these sentences such that this interaction could make sense semantically, and (iii) “translate” these sentences into a musical duet.

Until now, we have seen how the quotations associated with the category of “building musical connections” revealed how the online learning environment designed for the study can give rise to positive outcomes in terms of developing meaningful relationships with the instrument and the group. In both cases, it is arguably the creative, explorative activity that learners experience as they play and collaborate with others that makes this possible. In what follows we expand on these themes to focus on how physical movements and technical exercises contribute to the overall learning experience of the students.

### Control and Creativity

#### Moving to Create

As mentioned earlier, physical movement is an essential aspect of how musical skills can be acquired and developed. The mastering of adequate (e.g., breathing) techniques, fingering styles, and posture, for instance, is certainly central to a musician’s learning trajectory. And therefore, these elements are often seen as building blocks for novices. But while an initial focus on control and fine motility can be indeed beneficial in many ways, it is not always experienced positively by the students:

“At the beginning […] there was more emphasis on control, but over time I noticed that I did not like that control. […] For example, when my clarinet started playing with a ‘squeak’ in the beginning, that did cause frustration. But I notice that the more frustration, the more this ‘squeak’ came in. So basically: the more control I wanted to have, the less good what I wanted to play was. So, at some point I just let it go, just started focusing more on a natural posture while blowing, and it improved a lot. I actually think it’s about that less control to get more out of it.” (3, B)

When asked to provide a further example, the same participant comments as follows:

“Holding the clarinet forces the body into a certain position. You have to hold it, and the arms and the fingers are in a certain way. [The instrument] also has a certain weight, so it’s going to direct the movement. For example, I move a little bit slower. So, on that level, there’s also a certain ‘physical respect’ towards the instrument.” (3, B)

Playing an instrument, in other words, involves dealing with associated physical constraints, which may limit our natural inclination to move. This leads to a number of challenges, to which each learner may respond differently. For such a reason, one of the main ideas behind the study was to stimulate more freedom through larger bodily movements (e.g., doing “step exercises”^6^ while playing, or generating a new musical pattern while moving only certain parts of the body such as leg or an arm). As reported in the following quote, this can have a significant impact on how we engage with the instrument:

“Through bodily movement…your breathing becomes different, and you also focus more on what you are doing. Movement in itself was never difficult for me.” (1, B)

**Figure 4:**
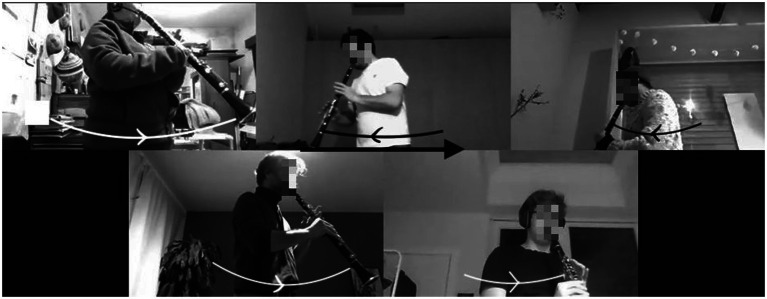
Playing a musical pattern while using lateral movements of the upper body. The arrows show the direction of the movement.

The key role of the body is thus evident at initial phases of the course, where learners were asked to imitate a rhythmical pattern (long–short–short) with a specific way of walking (slide–step–step) or moving the upper part of the body. An illustration of such an exercise is offered in Figure 4.

The positive outcomes emerging from such an exercise and more general comments about the role of movements are expressed in the following two quotes by the same participant:

“I often notice that certain aspects you have to study, like rhythm, are much easier if you link something physical to it. Those steps are a sort of support for me, just like the ‘slide, step, step’ exercise. Then I know that if I follow that pattern or do it that way, then I have to keep going for that long. […] In that way, I also find that movement very useful, as a kind of, […] metronome. I think that is the best comparison, because the coupling between body and instrument comes out best that way, a kind of accompaniment.” (3, A)

“with the long notes, for example, I also notice that, when I move, I hold on to a note until I change movement. Specifically, for example, I play the do when I put my left foot, and then I play it until I put the right foot. This is then purely rhythmical. If I then speed up the movement, that also logically links to a much faster alternation [and] in the tonguing [technique]. […] And then I try to pay attention to the fact that it’s not the clarinet that controls the body. So not the body following the clarinet, but the other way around, the clarinet following the body.” (3, A)

This last point suggests that with less constrained movements, the sense of control felt when interacting with the instrument can be significantly impacted, even at the very beginning of one’s musical journey. This also emerges when considering the emotional aspects that music-making activities often entail. In the following quote, another participant refers to the “kinematic musical task” proposed by the teacher, which consisted in creating a short choreography based on *Laban motif notation*^7^ (see Hutchinson Guest, 2007) that involved the creation of a new melody.

“Sometimes I had the feeling, especially during the second exercise in which we had to compose something ourselves with movement and melody, that I could put my emotion, ‘my thing’ into it.” (2, B)

Despite the challenges of not being able to move together in a “real” space, and not having a close guidance by the teacher (as it would be in a more formal context), the course offered a number of movement-based resources that learners could use to take responsibility for their own learning and improve their musicality. In what follows, we take a closer look on how this impacted the overall quality of their learning experience, and their motivation playing the clarinet.

#### Creative Potential

One of the aims of the musical course, we designed was to inspire students take more responsibilities for their own learning. In a sense, this involves helping participants discover their creative drive, enhancing meaningful interactions with the instrument and the group. These two aspects, as we have emphasized all along, are intrinsically linked with each other. An example of how this is experienced by students emerges from the following two quotes by the same participant after lesson 4 and lesson 12, respectively:

“I do not really have the fantasy to start playing all kinds of things myself and to figure it out. I have that to a lesser degree, I notice that if you give me something like ‘learn to play that’, then I can do that perfectly well. But if you say to me ‘create something yourself’. I cannot do that.” (1, A)

“You do creative things because you are challenged to work together and do things in that regard. […] So, the interaction with each other does make you be creative. You do get lured out of your comfort zone to do something.” (1, B)

The bonds between participants that emerged in the course, it can be argued, played a key role in fostering creative thought and action despite the physical distance. However, it is also the experience of playing music in itself that contributes to musical and creative flourishing:

“for me, learning to play the clarinet is a kind…that’s a difficult…an enrichment anyway. You can connect a lot of things to it. For example, in my spare time I do a lot of research, I read a lot of things. And then you notice that I can connect certain stories or certain ideas to it, and that they come back.” (3, A)

This capacity to make novel associations is often regarded a central aspect of creative cognition (see Benedek et al., 2012) and is indeed reported in the previous quote. Remarkably, this does not only involve connections between different domains of experience (e.g., reading stories and playing music, as in the last statement), but also speaks directly to how one can learn music.

“One of the recurring thoughts is whether I’m playing it right, whether the sound is correct. Especially when I’m practicing, for example the scales, I often think about whether the sound is correct, whether I blow in the right way. On the other hand, suppose I start practicing a little looser and then play more with the clarinet, then those thoughts are less, and then I can afford to make a mistake or play it in a different way. Yes, in that case you can also put a little emotion in there by just using a different sound or a mistake. It’s very different though. If I’m really practicing, then I often look at—I would say it’s almost normative—what is right or wrong. But when we do creative exercises, it’s completely different.” (3, B)

The issue of “control” discussed earlier makes again an appearance. When engaging in creative exercises, such as developing together new melodies one after the other (such that each one continued from where the last one finished), there appears to be less control overall, not only in terms of physical constraints but also regarding what musical aspects can be developed. For example, in the last statement, the participant mentions how mistakes could be thought of as musical opportunities and more emotional nuances could be used to explore such opportunities. Engaging in such creative discoveries, however, might be not enough to “feel” creative, as creativity is often associated with the mastery of certain mental or technical skills, which one can manipulate to achieve novel, valuable results. This is made explicit in the following quotes by two different participants:

“You can be creative with a bike, but you cannot be creative with your bike if you cannot ride a bike properly.” (2, B)

“I cannot say of myself that I am creative […], because I think you can only be creative when you have a certain skill or mastery. […] And I think […] of music in itself as a very creative thing. If you try to explore that, I think you are being creative in some way, but separate from that mastery and so on. I find that very difficult, because […] I think that you actually have to master something before you can start being playful with it, and only then…It’s kind of, a little bit anyway, an expert level.” (4, B)

Yet, when asked to elaborate more on this, this last participant makes a further important point:

“I have always considered creativity to be a bit of an expert-level [thing]. Whereas now I’ve noticed that music in itself can also be a creative method.” (4, B)

By using the term “creative method,” the participant arguably points to the capacity of music to enhance the creative potential of the individual, echoing previous statements concerning the “connections” one can come up with when learning music, even at the earliest phases.

## Discussion and Conclusion

The present research explored the verbal descriptions and reflections of participants who enrolled to a group, online course based on creativity, movement, and collaboration. As such, this work complements other studies that focus on the transition between face-to-face and remote learning (e.g., Camlin and Lisboa, 2021; Ritchie and Sharpe, 2021), on online music lessons offered to more skilled participants (e.g., Johnson, 2017), and on forms of musical learning based on imitation and the reproduction of scores (Lisboa et al., 2005; Hanken, 2017). To achieve our objective, we asked four novices to participate in a 12-week, newly designed music course delivered remotely from the start and conducted two semi-structured interviews with each learner after 1 month and 3 months from the beginning of the course. A thematic analysis of the qualitative data gave rise to two main themes, that of “Building Musical Connections” and of “Control and Creativity,” respectively.

Regarding the former category, two main codes were individuated: “Knowing the Instrument” and “Knowing the Group.” As revealed by several statements reported during the first interviews, participants were perhaps not entirely sure if the online setting of the course, the main instrument, and the collaborative approach offered, would work for them. However, all participants were generally satisfied after the last interview and motivated to keep playing in the future. As such, the partnerships developed by each participant with group and instrument can be thought of as valuable tools that contribute to musical skill development.

When looking at the instrument in more detail, a main motive emerged in the interviews: participants were generally able to create a strong bond with the instrument after an initial period of adaptation. This aligns well with the literature suggesting how tools and musical instruments can become, in a sense, “incorporated” through experience and practice, being treated as if they were part of the musician’s cognitive system (Nijs et al., 2013; Simoens and Tervaniemi, 2013; Rojas, 2015; Nijs, 2017). By acting “creatively” with it, new musical possibilities are arguably discovered directly through the musical instrument, without recurring to prior thought or conceptual preparation (see Borgo, 2005, 2007). There is thus a synergetic “dialogue” that can develop between instrument and musician—a possibility captured by one of the participants when s/he describes the clarinet as a “friend.”

The description of how participants engaged with the group was equally fascinating, as it emphasizes the importance of creating a safe learning environment, despite the physical distance. This echoes existing work that shows how pedagogical settings based on mutual trust, open discussion, and collaboration between peers can provide an optimal frame for skills to be developed (e.g., Robinson and Kakela, 2006; Clapper, 2010), in both formal and informal musical contexts (see Lebler, 2008). Remarkably, the interviews revealed how participants embraced the ambiguity of “distant” social relationships to interact constructively through a process of progressive attunement with the musical instrument, which involved a creative framing. This was particularly evident when participants mentioned how the “breakout rooms” gave them more space for freedom and interaction, in turn providing them with more shared responsibilities for their own learning. Among others, the importance of shared responsibilities for musical development in novices has been recently emphasized in an empirical study by Schiavio et al. (2020b), where it was demonstrated that peer-learning techniques based on synchronization and turn-taking gave rise to more accurate musical outcomes when compared to imitation, highlighting the key role of collaborative contexts where both learners are given equal responsibilities in the concrete act of playing music.

A combination of factors involving creativity and collaboration emerged in several of the statements reported under the second main category, that of “Control and Creativity.” Here, a number of verbal descriptions can be seen to explain how specifically musical and non-musical (e.g., walking) movements were creatively used by the group during the course. Let us begin with the first code of this category that is “Moving to Create.” Quotes placed under this header highlight a tension between the physical constraints inherent to what playing musical instruments entail and the freedom of movements necessary to let creative ideas and action flow with ease. It should be noted that examining the reciprocal interplay between the opportunities offered by the physical (and the social) environment and the capacity to generate creative (e.g., musical, artistic, etc.) outputs a key theme in recent work on creative cognition and ecological dynamics (see, e.g., Kirsh, 2014; Malafouris, 2014; Orth et al., 2017; Kimmel and Rogler, 2018; Schiavio and Benedek, 2020). The qualitative insights reported in our study contribute to this line of work with a specific focus on music, not only emphasizing how moving freely while playing can facilitate certain musical activities (e.g., the rhythmic exercises with steps, moving along with a chord or stick, and improvising music while freely moving with the upper body), but also how it can help develop a more personal relationship with the clarinet, where emotional aspects can be effectively put into play in a creative fashion (see again Nijs, 2019).

This last insight brings us to the second code emerged under the present category: “creative potential.” Here, participants described how their capacity to develop novel, surprising, and valuable associations of ideas and (musical) actions can be enhanced by the collaborative and movement-based dimensions of inherent to the course. While this remains to be further investigated, there is already a wealth of research that shows how walking can boost creative ideation (Oppezzo and Schwartz, 2014), and that recognizes the structural link between social interaction and creativity across a range of contexts in music and beyond (see, e.g., Sawyer and De Zutter, 2009; Burnard and Murphy, 2013; Glãveanu, 2014; van der Schyff et al., 2018; Verneert et al., 2021). Again, the multimodal connections between creative, social, and movement-based activities offered in the course are seen to positively shape the participants’ learning trajectory, helping participants feel of themselves as more creative, as well as develop meaningful relationships with the group and the instrument itself, despite reasonable initial concerns.

In all, because our participants were generally positive^8^ when describing their learning experience, the present exploratory study suggests that a cause of the learning issues reported in the recent literature in music education (e.g., when describing remote learning during the pandemic) might in fact be found in the *sudden shift* in pedagogical methods caused by the pandemic, rather than in remote education *per se*. This last insight aligns well with research that shows that music teachers may find the systematic use of technology for their didactics highly problematic when not adequately supported by their school, for example, through training sessions (see Gall, 2013; Schiavio et al., 2021a). Such an observation, as anticipated, is particularly relevant when considering the recent health emergency caused by the spread of SARS-CoV-2 around the globe, as a result of which many educational offers had to be radically transformed. For those pedagogical activities based on music, the shift was particularly difficult, given the key role of close interactions between students and teachers (Johnson and Merrick, 2020; de Bruin, 2021). As learning and doing music online present several challenges that have been addressed in many contributions (see, e.g., Baratè et al., 2020; Biasutti et al., 2021), gaining a richer insight into the lived experience of novices who start learning to play an instrument through this format can be particularly helpful to better individuate and face such challenges.

Several differences may arise when music lessons are offered remotely rather than in person, including the type of personal relationships that develop between group members (which lacks a more immediate dimension) and the absence of key musical and social aspects such as touch and gaze (which can be particularly useful when dealing with specific technical problems; see Antonini Philippe et al., 2020). Facilitating body movement, among others, could become a major obstacle for the teacher; the latter—without physical proximity and adequate training with technology—could struggle to find a way to promote the development of convincing motor solutions for the students. As such, the lack of “real” closeness can be a disadvantage for students as well, who might need more time to musically “attune” to the group and to the teacher.

Participants of our study nevertheless developed an important sense of togetherness as they were given various responsibilities and were invited to creatively explore different body configurations alone or together. As such, it is suggested that both remote and in-person modes of learning can be approached in a positive way, fostering synergies that involve a reciprocal interplay of creative, interactive, and bodily dimensions. We are not claiming that the style of teaching illustrated in this paper would work better than it would have in an in-person setting: Our participants did not have any previous experience with other learning approaches in music, which makes a direct comparison hardly accomplishable. That said, the positive outcomes of this study could be taken as an opportunity to reflect on the value of technology-enhanced pedagogies. Not only is this point particularly important amidst the times of global crisis we are currently experiencing, in which physical interaction is being increasingly replaced with virtual connections; it also points to the possibility that similar learning programs might be implemented when the global health emergency will be over. Indeed, we suggest that a remote setting can also facilitate social inclusion in contexts (i.e., marginalized communities) where attending musical lessons privately might be difficult.

Before concluding we wish to note that, as a qualitative study with a small sample size, the present research features one important limitation, that is, the lack of generalizability. But while our data cannot be used to test general hypotheses or theories, they can still provide a comprehensive window on the lived experience of musical beginners. And, as such, our findings may inspire novel pedagogical approaches in music that wish to offer novice learners a remote context where creativity, interaction, and freedom of movement can be fostered. Another limitation concerns the rather short period (3 months) of lesson, which may seem to hinder an assessment of instrumental training. We acknowledge the value of a more longitudinal approach (which could also illuminate on how specific motor configurations develop individually and in group), yet the goal of this study was not to *assess* musical training but rather to gain insights on how musical novices would experience their first musical course in a remote setting with a special focus on the role of creativity, interaction, and bodily movement. Moreover, the very first months of a novice’s learning path are a particularly interesting period of investigation, as so many aspects of music-making come together, often leading to a focus on technical aspects at the expense of creativity and expressiveness. It should also be said that our participants were adults, so things could have gone differently in the case of younger students (even where the latter could feel more comfortable in a totally virtual environment).

Finally, it could be argued that our participants might be biased toward offering a positive evaluation of the course, because they were self-selected volunteers, and because teacher and interviewer were the same person. We acknowledge this possibility, being reminded by Seidman (1998, p.35) that “the teacher-researcher should seek to interview students in some other setting *with some other teacher who is using a similar method or curriculum*” because “a student can hardly be open to his or her teacher who has both so much power and so much invested in the situation.” However, given the uniqueness of our pedagogical approach, such a teacher was not available. Also, Seidman’s concerns seem to refer mainly to a teacher-centered context. A more student-centered approach, on the other hand, may foster an environment in which pupils feel safer to express their concerns, ideas, and opinions. This latter was the approach of the present study, whereby the teacher–researcher developed a familiarity and closeness with the participants uncommon in other types of research, allowing students to express their thoughts freely and critically during the lessons and the interviews. Indeed, the teacher-researcher has a privileged position characterized by “the vicarious experience of having been there” (Merriam, 1998, p. 238) and allowed “weaving together the successes, the failures, and the tensions involved with the innovative approach” (Luke, 2004).

To conclude, we believe the preliminary findings reported in this contribution may spur teachers and researchers to further explore and develop new learning paradigms in instrumental music teaching settings that more strongly cultivate the creative potential of groups and individual learners, that place considerable focus on free movement and expressivity from the very beginning, and that stimulate collaborative and peer-to-peer learning across different musical genres and personal styles. Among other things, a way to extend the present research might involve a focus on video data and quantitative approaches. In a partially similar way to existing interdisciplinary work (see, e.g., Bietti and Baker, 2018; Olivares and Cornejo, 2020), a detailed analysis of the video recordings of the lectures could help us gain more detailed information on how certain motor configurations develop over time, reflecting the growing interest in musical research for the study of the bodily and kinesthetic aspects of experience. A quantitative methodology guided by specific hypotheses could in fact integrate the qualitative findings reported in this paper, offering a privileged way to examine specific motor variables (such as breathing or fingering) fundamental for the acquisition of musical skills, further enriching our understanding of how novice musicians thrive from their first musical steps in a remote setting.

## Data Availability Statement

The anonymised data supporting the conclusions of this article will be made available by the authors, without undue reservation.

## Ethics Statement

Ethical review and approval was not required for the study on human participants in accordance with the local legislation and institutional requirements. The patients/participants provided their written informed consent to participate in this study. Written informed consent was obtained from the individual(s) for the publication of any potentially identifiable images or data included in this article.

## Author Contributions

Designed the study: AS and LN. Collected the data: LN. Analysed the data: AS and LN. Wrote the first draft: AS. Edited the manuscript: AS and LN. Both authors have made a substantial, direct and intellectual contribution to the work, and approved it for publication.

## Funding

AS acknowledges the support of the Austrian Science Fund (FWF). This research was funded by the Austrian Science Fund (FWF), project number: P 32460. LN acknowledges the support of the Flemish Research Fund (FWO), project number: V402921N.

## Conflict of Interest

The authors declare that the research was conducted in the absence of any commercial or financial relationships that could be construed as a potential conflict of interest.

## Publisher’s Note

All claims expressed in this article are solely those of the authors and do not necessarily represent those of their affiliated organizations, or those of the publisher, the editors and the reviewers. Any product that may be evaluated in this article, or claim that may be made by its manufacturer, is not guaranteed or endorsed by the publisher.

## Footnotes

^1^We considered our participants “musical novices” as they reported that they never formally or informally learned to play a musical instrument (including voice).

^2^A haiku is a style of poetry from Japan that features very short lines and that usually evokes natural imagery.

^3^In addition to such collaborative practices, the teacher could also use the breakout rooms to invite students and work individually with them on a specific aspect of their playing.

^4^This orientation can be seen in any form of dualism that separates mind from matter.

^5^ https://flipgrid.com

^6^These were characterized as follows: (1) students perform a specific stepping pattern in a loop: right foot to the right, left foot joins, left foot to the left, and right foot and (2) the rhythm of a song (e.g., short-short-long) is translated in a stepping pattern (e.g., step-step-slide).

^7^Laban motif notation is a simpler version of Laban notation, consisting of a series of symbols that, when combined, express a single moment of movement.

^8^Notably, three out of the four participants continued to follow lessons in formal and informal lessons.
