# Supplementary material for: Implementation of a Remote Instrumental Music Course Focused on Creativity, Interaction, and Bodily Movement. Preliminary Insights and Thematic Analysis
Source: Front Psychol. 2022 May 20;13:899381. doi: 10.3389/fpsyg.2022.899381 (PMC9165424; doi:10.3389/fpsyg.2022.899381)
Supplement: Supplementary file 1 [file Table_1.DOCX]

|  | **Question**   - **subquestions** |
| --- | --- |
| 1. | You have recently started to learn playing the clarinet. In general, how did you **experience** this so far? Try to describe your new musical experience in detail. |
|  | - What **physical** and **emotional** sensations accompany your experience of learning music? |
|  | - What do you think about when playing the clarinet? “For me, learning the clarinet is….” (please complete the phrase, use only one word) |
|  | - Is there a difference between the **lesson and practicing** at home? Please explain. |
|  | - Do you feel in control of your **musical development**? |
|  | - How do you organise your **practice** sessions? |
|  | - Do you follow the practice sheets? Or do you rather repeat activities you remember from the lesson? |
|  | - Do you use the information on the **website**? |
| 2. | Can you tell something about your **relationship**, or your **connection,** to your instrument? |
|  | - How do you experience the **physical** connection to your instrument? |
|  | - How do you relate **emotionally** to your instrument? |
|  | - What do you **think** about your instrument? To me, the clarinet is… (see before) |
|  | - Do you have the feeling that you **control** the instrument? |
|  | - How do you experience the combination of moving and playing? Does it affect your connection with the instrument? |
| 3. | Do you feel you can be creative when learning **the clarinet**? Please explain |
|  | - Are there **specific moments** where you have the feeling that you are less or more creative? |
|  | - Please complete the following sentence: “for me being creative while playing is….” |
| 4. | How do you experience the **group interaction** during the lesson? |
|  | - Do you have the feeling that you can participate in the others’ learning? How? |
|  | - On flipgrid there is not yet real interaction between the 4 students. What do you think is the reason for that? |
| 5. | What are the aspects of the lessons you consider most **interesting** for learning music? Why? |
|  | - What aspects of the lesson do you think contribute the most to your learning process? |
|  | - Can you tell me something (more) about the use of movement in the lesson and at home? |
|  | - Can you tell me something (more) about the use of technology in the lesson and at home? |
| 6. | Would you like to add anything else? |
